# Dendritic Cells in Innate and Adaptive Immune Responses against Influenza Virus

**DOI:** 10.3390/v1031022

**Published:** 2009-11-24

**Authors:** Artur Summerfield, Kenneth C. McCullough

**Affiliations:** Institute of Virology and Immunoprophylaxis, Sensemattstrasse 293, CH-3147 Mittelhäusern, Switzerland

**Keywords:** dendritic cells, influenza, innate and adaptive immune responses

## Abstract

Dendritic cells (DC) are major players in both innate and adaptive immune responses against influenza virus. These immune responses, as well as the important interface between the innate and adaptive systems, are orchestrated by specialized subsets of DC, including conventional steady-state DC, migratory DC and plasmacytoid DC. The characteristics and efficacy of the responses are dependent on the relative activity of these DC subsets, rendering DC crucial for the development of both naïve and memory immune responses. However, due to their critical role, DC also contribute to the immunopathological processes observed during acute influenza, such as that caused by the pathogenic H5N1 viruses. Therein, the role of different DC subsets in the induction of interferon type I, pro-inflammatory cytokine and chemokine responses is important for the outcome of interaction between the virus and host immune defences. The present review will present current knowledge on this area, relating to the importance of DC activity for the induction of efficacious humoral and cell-mediated immune responses. This will include the main viral elements associated with the triggering or inhibition of DC activation. Finally, the current knowledge on understanding how differences in various vaccines influence the manner of immune defence induction will be presented.

## 1. Introduction

During the last 25 years, influenza A virus (IAV) has served as an important model to understand and dissect the essential roles played by dendritic cells (DC) in immunity against virus infections. This applies to both innate immune responses – including interferon (IFN) type I, other cytokine, and chemokine secretion – and the development of adaptive immune defences – including CD4 helper T-cell (Th), CD8 cytotoxic T-cell (Tc) and antibody responses. Here we review the current knowledge in these areas, and highlight the value for translational research towards improving human vaccines against influenza as well as the important gaps in this knowledge.

## 2. Innate immunity

### 2.1. Importance of DC responses in innate immunity

IAV has been extensively described as a model virus to study DC involvement in innate immune responses. Effective innate immune responses are important in terms of the variety of defence functions mediated: (i) Limiting early virus replication prior to adaptive immune defence development, particularly through secretion of cytokines with antiviral activity, such as type I IFN (ii) recruiting by DC-derived chemokine activities of inflammatory cells and additional DC to the sites of infection, which enhance the innate responses including phagocytosis of apoptotic and necrotic cells containing virus; (iii) release of IFN and other cytokines, which in turn provide alarm signals to the immune system for activating cells of both the innate and adaptive systems, such as NK cells and cytotoxic T cells. In addition to the innate defence activities, DC are also involved in promoting adaptive responses, for which the elements (ii) and (iii) above are particularly essential. Indeed, it is this dual function of DC in mediating innate responses and acting as professional antigen presenting cells that provides the linking of innate and adaptive immune responses. These general aspects of DC biology have been reviewed more extensively by others [1–3]. The present section will focus primarily on the innate defences, with the adaptive immunity being considered later in the review.

IAV models (together with other viral models) have demonstrated that DC represent an important family of cells promoting antiviral innate immunity. Therein, an important contributory element is the specialized expression of receptors designed to respond to alterations in the environment with the cells have contact. Particularly important are the Toll like receptors (TLR) such as TLR3, TLR7, TLR8 and TLR9 involved in sensing viruses [4]. In this context, one realizes the significance of the two main functional specialized DC subsets – the so-called conventional DC (cDC) and plasmacytoid DC (pDC). The existence of these subsets appears to be widely conserved and a fundamental element in mammalian DC biology and response against viruses [5,6]. While the so-called cDC represent what can be termed classical DC with respect to their main function being antigen presentation, the pDC are distinctive in terms of their main function being as professional IFN type I-producing cells, with a particular ability to sense viruses [5]. This functional specialization of pDC relates to their constitutive expression of high levels of TLR7 and TLR9 as well as of IRF7, the most potent transcription factor for IFN type I production [7]. Furthermore, pDC possess important endocytic properties such as the ability to retain RNA and DNA in the TLR-containing endosomes, which permits a better interaction of the viral nucleic acid with the TLR and the signalling molecules Myd88 and IRF-7 [8]. In contrast, cDC respond to IAV in a TLR-independent manner [9], and sense influenza virus primarily via the helicase retinoic acid inducible gene I (RIG-I), representing a cytosolic RNA receptor [10].

### 2.2. DC responses to influenza A viruses

IAV can infect DC, leading to the synthesis of viral proteins. The infection is usually abortive in terms of infectious progeny generation, and non-cytopathogenic [11], although H5N1 viruses have been described to replicate in human and mouse DC yielding a cytopathic effect [12,13]. Often, IAV infection of both cDC and pDC activates the cells, and the maturation process is induced. This is seen in terms of co-stimulatory and MHC molecule upregulation, as well as CCR7 synthesis, a key chemokine receptor for DC migration to lymphoid tissue [11,14]. Nevertheless, as seen with the overview of this topic in Table 1, the degree of activation and the type of response induced is very much dependent on the IAV strain selected, the species under study, the exact subset of DC analyzed. Moreover, the type of virus preparation (e.g. purified, egg- or cell-derived) will have a strong influence, as will the treatment of the virus preparation (e.g. live, UV-, chemically or heat inactivated). Finally, the dose of virus applied to the DC is also critical with respect to the outcome of that interaction. Consequently, it is not surprising to find that results in the literature can present apparently conflicting conclusions.

### 2.3. Relative roles of different DC subsets responding to influenza A viruses

As with many (or even all viruses), IAV has the capacity to counter innate immune responses in its primary target cells, represented by epithelial cells. A main element therein is preventing the induction and action of type I IFN, effected primarily through the functions of the viral non-structural protein NS1 (for recent reviews see [15,16]). This type of inhibition is also active in IAV-infected cDC. In contrast, pDC are strongly activated by IAV despite the presence of NS1, resulting in induction of inflammatory and IFN responses (Table 1). Considering that pDC are an important source of not only IFN type I, but also proinflammatory cytokines and chemokines [17] (Table 1), it could be proposed that pDC assure innate immune responses, certainly against influenza viruses. Stimulation of pDC requires IAV binding to sialic acid receptors and an intact endocytic system (unpublished results, [18]). Whether fusiogenic activity is required remains unclear, but denaturation of the virus by heat treatment (>60°C) destroys its interferogenic properties (own unpublished results using porcine pDC). Studies with mouse pDC have demonstrated that the actual activation of the pDC likely occurs through TLR7-mediated recognition of single-stranded RNA [19], and is independent of virus replication [20]. In support of this concept, it has been shown that influenza virus-derived virosomes cannot activate pDC (own unpublished results, [21,22].

Murine studies have indicated that pDC provide a major contribution to IFN type I responses during influenza [23]. In particular, pDC would contribute to early IFN responses in lymphoid tissue as a consequence of their presence in these organs, as well as their constitutive expression of IRF7 [24]. This IFN can also promote a positive feedback in the respiratory tract in terms of IRF7 induction, thus licensing non-pDC including cDC and epithelial cells for IFN type I production [24–26]. On the other hand, *in vivo* studies with mice have demonstrated that the respiratory epithelium also represents an important source of IFN type I [23]. These studies relate to two recent reports performed in mice suggesting that pDC are dispensable for protective immunity against influenza [27,28]. However, it is uncertain whether this will also apply to natural hosts of IAV such as man and pig. One reason to doubt this is that the laboratory strains used for the above studies have a defective Mx gene. The Mx system represents an important response mechanism to type I IFN-dependent signalling, and confers protection to mice against IAV infection [29]. In fact, Mx-competent mice are even resistant to the 1918 H1N1 and highly pathogenic H5N1 viruses [30]. It could thus be speculated that in Mx-compentent mice the pDC-derived IFN could have an important contribution to protective antiviral innate immune responses.

In contrast to pDC, cDC respond only to live IAV, and this leads to a classical profile of DC maturation, together with the release of proinflammatory cytokines and chemokines (Table 1; for example [14,18,31]). The release of chemokines from infected human DC occurs in three waves following an apparent program to recruit effector cells of the innate immune response, followed by effector memory cells and naïve B and T cells [17]. It is important to note that the influenza virus NS1 protein can also downregulate this cDC activation and maturation. Wild-type IAV infection of cDC induces only a relatively modest cytokine production. In contrast, NS1 deletion mutants (ΔNS1) are potent activators of both mouse and human cDC, promoting DC maturation and induction of IFN, other cytokines and chemokine responses [32,33]. Due to these characteristics, ΔNS1 viruses have been proposed as promising candidates for a live vaccine [34,35].

## 3. Adaptive immunity

### 3.1. Importance of DC for induction of CD4 and CD8 responses

It is well established that DC play a major role as professional antigen presenting cells for priming of both CD4 Th and CD8 Tc lymphocytes [36]. For Th responses any protein antigen will be taken up by DC and at least in part be degraded in the endosomal pathway resulting in presentation through MHC class II. A critical issue of this presentation is the activation of DC which will promote antigen degradation and stable presentation of many MHC class II peptide complexes on the DC surface together with costimulatory molecules such as CD80 and CD86 [36]. Adoptive transfer experiments have demonstrated that such CD4 Th responses can protect mice from lethal challenge infection [37]. This protection required B cells indicating that the classical helper function of CD4 T-cells is important in protection against influenza, although functional diverse subsets of CD4 Th have been described including some with cytotoxic activity [38]. Early *in vitro* studies with mouse splenic DC indicated that DC infected with live virus stimulated both CD4 Th and CD8 Tc, whereas those loaded with inactivated virus tended to induce only CD4 responses [39]. However, a later study with human DC demonstrated that inactivated virus with intact fusiogenic activity could restimulate Tc [40]. This relates to the necessity of antigenic peptide to access the MHC class I presentation machinery, which can be by de novo synthesis of viral proteins or by cross-presentation of endocytosed antigen. Cross-presentation can be mediated by transfer of antigen to the cytosol, delivery to the proteosome and the ER. Alternatively, MHC class I molecules get access to the endosomal compartment. There are many pathways controlling such processes, which have been reviewed extensively elsewhere [41,42]. In principle, theses processes depend on the type of receptor used for endocytosis of the antigen, the activation state of the DC and the functional specialization of the DC subset (see below). With respect to conventional influenza vaccines these pathway are is still not sufficiently exploited as only live vaccines seem to efficiently promote influenza specific Tc responses [43].

### 3.2. Involvement of respiratory DC in promoting T-lymphocyte responses

More recent studies performed with murine *in vivo* models have been focusing on the role of various DC subsets – summarized in Table 2. Clearly, both cDC and pDC have the ability to stimulate naïve CD8 T cells and cross-present antigen [14,44]. Zammit *et al.* [45] have even demonstrated an important role for cDC in restimulation of Tc memory responses *in vivo*. More detailed studies on various subsets of respiratory DC (RDC) in murine models have demonstrated a distinct functional specialization. Of particular importance for protective immunity against IAV are the intraepithelial DC present in the trachea and the conductive airways. These intraepithelial RDC, which in the mouse are CD11b^−^CD11c^+^CD103^+^Langerin^+^, are present under steady state conditions. Following IAV infection, they rapidly migrate to the draining mediastinal lymph nodes (MLNs), where antigen presentation and induction of adaptive immune responses occurs [27,46]. This relates to the observation that intraepithelial RDC are efficiently infected by IAV, both *in vitro* and *in vivo*, and thus carry antigen to the MLN [46,47].

A second population of RDC in the submucosa of conducting airways are identified as CD11b^+^CD11c^+^CD103^−^ in mice. These cells increase significantly in number during influenza, and also become positive for the viral nucleoprotein [27,46,48]. However, the published reports are in disagreement about their actual role in antigen presentation, which may reflect the virus stains being employed and the use of mouse models for the analyses.

Finally, there are various subsets of DC in the interstitium of the lung. These include pDC as well as resident cDC subsets. In addition, under inflammation there is a prominent increase in the number of monocyte-derived inflammatory DC. The latter apparently participate in inflammatory reactions, and promote CD8 effector T-cell recruitment and proliferation [49], although they do not seem to be involved in antigen presentation within the MLN [27,46]. There are several studies demonstrating that the resident MLN DC present IAV antigen, promoting CD8 T-cell responses (in the mouse, these are defined as the CD8α+ DC subset) [50,51]. It has been proposed that this is at least in part mediated by antigen derived from migrating RDC, in particular the intraepithelial RDC subset [52,53]. While preparing this manuscript another review giving more detail on RDC was published [54].

### 3.3. Involvement of pDC in promoting T-lymphocyte responses

In addition to the above studies on the various cDC subsets, there has also been work on the role of pDC in promoting adaptive immune responses. Attempts to identify the contribution of pDC have employed antibody-based depletion studies in mice. However, they were unable to define any prominent role for this DC subset in CD8 T-cell priming during influenza [27]. It should be noted that pDC can promote the maturation of cDC necessary for efficient antigen presentation and therefore activation of adaptive immune responses. An absence of an apparent role for pDC in development of Tc responses might imply that this “cDC maturation-inducing” activity is not an absolute requirement. However, one has to remain cautious when interpreting results from one species as being applicable to other species. This is particularly the case with mice, wherein the responsiveness of cDC and pDC may show distinctive characteristics when compared with that observed for human and porcine cDC and pDC [6,55,56].

### 3.4. DC and the induction of antibody responses

Considering the essential role played by DC in inducing antigen-specific T lymphocyte responses, it is evident that DC would contribute indirectly to B-lymphocyte responses through the activation of Th-cell responses. In contrast, there is no information concerning any direct interaction of DC with influenza virus-specific B-cells. Moreover, DC depletion using the CD11c-diphteria toxin receptor mouse model indicated no contribution of CD11c^+^ DC to antibody responses [27]. This study did show that pDC were important, in that their depletion reduced the levels of antibody induced. Previous work had demonstrated that IAV-activated human pDC can promote plasma cell differentiation, in which pDC production of IL6 and IFN-α were important [57]. This role of pDC was confirmed indirectly using a MyD88- and TLR7-knockout mouse model. Mice lacking these molecules, which are essential signalling elements for pDC activation and IFN-α release, had a defective isotype switching [58]. This also applies to the immune response induced by a whole virus inactivated influenza vaccine but not to split virus or subunit vaccines in mice [59].

## 4. Immunopathology

### 4.1. Roles for DC in influenza virus-induced immunopathology

Immune responses have the character of a double-edged sword, on the one side controlling immune defence development and homeostasis, on the other side contributing to immunopathological phenomena when over-activated or under loss of control. The latter is a relevant area to virus infections which interfere with aspects of immune defence regulation. Accordingly, it would not be surprising to find a role for DC in immunopathological reactions associated with IAV infections. Certainly, aberrant cytokine responses have been proposed as one of the triggers leading to fatal infections in humans with H5N1 viruses [60,61]. Such reactions are likely mediated by overproduction of IFN type I together with proinflammatory cytokines or other inflammatory mediators. In this context, one needs to consider pDC, known to be the most potent producers of IFN-α as well as certain pro-inflammatory cytokines such as TNF-α and IL-6 [5]. As described above, such IFN-α will sensitize other cells including cDC and epithelial cells for additional IFN type I production, as well as release of other cytokines and chemokine [24–26].

### 4.2. The “cytokine storm” phenomenon

An excessive or imbalanced production of such cytokines and chemokines would interfere with the normal functioning and control of immune response developments and homeostasis, leading to immunopathological situations. However, it has not been possible to confirm such a role for pDC, at least in mouse models [27,28]. Furthermore, a general inhibition of cytokine responses in mouse models did not significantly alter the course of the disease [62,63]. This could question the concept of a role for cytokines in IAV-induced disease – the “cytokine storm” concept – but it may equally question the value of the mouse model for such studies. Certainly, IAV often requires adaptation to mice for studies in this model, or the use of particular mice strains (often with congenital defects). Moreover, a recent study using non-human primates has provided data supporting the “cytokine storm” concept [64]. H5N1 viruses are also known to be relatively more potent activators of human pDC compared with human IAV [65], at least *in vitro*. However, one should bear in mind that the “cytokine storm” alone is unlikely to be responsible for all aspects of IAV pathogenesis, and H5N1 fatality probably results from a combination of events following virus infection of the host [60].

### 4.3. Roles for DC other than the pDC

In addition to pDC, other DC such as monocyte-derived inflammatory DC, which accumulate in the lung during infection, are likely to participate in disease processes. This has been demonstrated in a mouse model for so-called TNF/iNOS-producing DC, which are of monocyte origin [49]. Also human monocyte-derived DC and blood myeloid DC are efficiently infected and even killed by H5N1 viruses at least *in vitro* [12]. This might provide indications relating to the virulence of such viruses. However, more work is required in this area to define the potential roles played by the different DC subsets, particularly those in mucosal sites of the body such as the various RDC, and also in species which are more natural hosts for IAV. It is likely that the different DC subsets will play particular roles during IAV infection and disease development. Their activities will in turn be influenced by events occurring *in situ*, including the activities of infected epithelial and endothelial cells, as well as inflammatory infiltrates.

## 5. Conclusions and implications for vaccines

The knowledge which has accumulated on the family of DC – their distinct anatomical localization, migratory properties, and specialized function in innate and adaptive immune responses – provides important insights to aid development of more efficacious and tailored vaccines. From mouse studies at least, it is evident that activation of CD4, CD8 and B-cell responses is ensured by specialized subsets of DC. Certainly, the triggering of innate immune responses by targeting pDC has the potential to promote T- and B-cell responses. This could partially explain the reports demonstrating higher potency for whole inactivated vaccines compared with virosomes and subunit vaccines, with respect to antibody induction [22,66]. One should also consider the potential value of targeting intraepithelial DC in the lung airways, which efficiently migrate to the draining lymph nodes for presentation of antigen and induction of adaptive immunity. Such targeting is important for promoting mucosal (intranasal) vaccination, which is currently lagging well behind in development compared with parenteral vaccination strategies. Studies with mouse models have indicated that cDC may not be crucial for the B-cell response against influenza. Of course, the open question is how this can relate to the role of cDC in other mammals more closely related immunologically to humans, or in which natural isolates of IAV can replicate and/or cause disease. If cDC are indeed less important for the direct activation of B-cell responses, one has to consider that a vaccine formulation should contain independent components targeting DC to promote effector T cell responses, as well as directly targeting B cells for efficient antibody responses. Both elements will be required for improved vaccines. This is an area necessitating elaboration if we are to promote more efficacious and better targeted vaccines against IAV. It is evident that translational research on the human counterparts of the different RDC, inflammatory migratory DC and the steady state lymph node DC is now required to identify their contribution in the immune response against influenza virus. With such information, one can progress rapidly with the possibilities of targeting these DC, and how best such targeted vaccines should be designed.

## Figures and Tables

**Table 1.** Innate immune responses involving DC induced by IAV.

	IFN-α/β		Pro-inflammatory cytokines		chemokines		maturation^b^	
	pDC	cDC	pDC	cDC	pDC	cDC	pDC	cDC
	
Live virus	++++	−/+^a^	IL-6, TNF-α, IL-12	IL-6 ^a^, TNF- α^a^, IL-12^a^	CCL3, 4, 22 CXCL10, 11, 13	CCL2, 4, 5, 8, 19, 22 CXCL9, 10, 11, 13^a^	+	+
ΔNS1 mutant	?	++	?	High responses	?	High responses	+	++
Inactivated virus^c^	++++	−	As live virus	−/+^d^	?	?	+	−

a low or undectable, potently increased after IFN-β priming

b measured in terms of MHC II, CD40, CD83, CD86 and CCR7 upregulation (dependent on the particular study)

c requires intact HA and fusiogenic activity

d conflicting reports

**Table 2.** Adaptive effector immune responses mediated by DC^a^.

	CD8^+^ Tc lymphocytes	Antibody
Intraepithelial RDC^b^	Potent priming	No evidence for a direct role
Steady-state submucosal or interstitial RDC	Conflicting reports	No evidence for a direct role
Monocyte-derived inflammatory RDC	Local expansion in lung	No evidence for a direct role
pDC	Conflicting reports	B-cell differentiation and isotype switching
Resident steady-state lymph node DC	Potent priming	No evidence for a direct role

a based on mouse models

b respiratory DC
